# Determination of 25‐Hydroxyvitamin D_3_ in Rat Brain by Derivatization‐Assisted LC/ESI‐MS/MS

**DOI:** 10.1002/bmc.70119

**Published:** 2025-05-22

**Authors:** Toma Shibuya, Fuwari Shishikura, Natsuki Yoshida, Shoujiro Ogawa, Tatsuya Higashi

**Affiliations:** ^1^ Faculty of Pharmaceutical Sciences Tokyo University of Science Tokyo Japan; ^2^ Faculty of Pharmacy and Pharmaceutical Sciences Fukuyama University Fukuyama Japan

**Keywords:** 25‐hydroxyvitamin D_3_, derivatization, LC/ESI‐MS/MS, rat brain, serum

## Abstract

Recent studies have suggested that vitamin D deficiency may have relations with various neuropsychiatric diseases as well as bone diseases. However, the concentrations of vitamin D metabolites in the brain and the relationship between their brain and serum concentrations remain poorly understood. To answer these questions, we developed and validated an LC/ESI‐MS/MS method for quantifying 25‐hydroxyvitamin D_3_ [25(OH)D_3_], an established marker for assessing vitamin D sufficiency/deficiency, in the rat brain and compared the brain concentrations with the serum concentrations. To enhance the assay sensitivity and specificity, the 25(OH)D_3_ was derivatized with 4‐[4‐(1‐pipelidinyl)phenyl]‐1,2,4‐triazoline‐3,5‐dione (PIPTAD) after purification of the brain sample by a two‐step solid‐phase extraction. A good linearity was obtained within the range of 20–1000 pg/g tissue, and the intra‐assay and interassay precision and accuracy were acceptable. In normal rats (*n* = 6), the brain 25(OH)D_3_ concentrations ranged from 128 to 175 pg/g tissue, which were extremely low (approximately 1/100) compared to the serum concentrations. The bile duct ligation caused the decreased serum 25(OH)D_3_ level, which produced the subsequent decreased brain 25(OH)D_3_ level (44–79 pg/g, *n* = 6). These results strongly suggested that the serum 25(OH)D_3_ concentration has a significant effect on its brain level.

## 1 Introduction

Vitamin D_3_, a major form of vitamin D in humans, is synthesized from 7‐dehydrocholesterol in the skin by the ultraviolet (UV) photochemical reaction followed by thermal isomerization as well as it is directly absorbed from one's diet. Vitamin D_2_, another form of vitamin D, is derived only from the diet, especially from vitamin D_2_‐containing supplements and sun‐dried mushrooms, and therefore, vitamin D_2_ and its metabolites are detected only in individuals taking these foods (Miyamoto et al. 2023). Vitamin D_3_ is first converted to 25‐hydroxyvitamin D_3_ [25(OH)D_3_], the circulating form, in the liver by vitamin D 25‐hydroxylase (Tuckey et al. 2019). The circulating level of vitamin D_3_ is much lower than that of 25(OH)D_3_ (Hu et al. 2020). 25(OH)D_3_ is then converted to 1α,25‐dihydroxyvitamin D_3_ [1,25(OH)_2_D_3_], which is the ligand for the vitamin D receptor (VDR), in the kidney by 25‐hydroxyvitamin D 1α‐hydroxylase (CYP27B1) to exert various biological activities including bone formation. Recent studies have suggested that vitamin D deficiency, which is diagnosed based on the decreased circulating 25(OH)D_3_ levels, may have relations with neuropsychiatric diseases including Alzheimer's disease, Parkinson's disease, schizophrenia, depression, and autism (Eyles et al. 2013; Rihal et al. 2022; Wassif et al. 2023), as well as well‐known bone diseases. However, at present, the concentrations of vitamin D_3_ metabolites in the brain and the relationship between their brain and serum concentrations remain poorly understood.

As the brain is encased in the skull, it is a good theory that the UV exposure‐based synthesis of vitamin D_3_ does not occur in the brain. The circulating concentration of 25(OH)D_3_ (*ca.* 40–100 nM) is nearly thousand‐fold higher than that of 1,25(OH)_2_D_3_ (*ca.* 40–150 pM) in humans (Higashi et al. 2010; Tuckey et al. 2019), and 25(OH)D_3_ also has a significantly longer half‐life than 1,25(OH)_2_D_3_ (2–3 weeks and 4–6 h, respectively) (Holick 2009). CYP27B1 and VDR are widely expressed in the brain (Eyles et al. 2005; Harms et al. 2011). When taken these observations together, a plausible theory is that the serum 25(OH)D_3_ migrates to the brain, then is converted to 1,25(OH)_2_D_3_ in a particular location of the brain to exert the effects on the brain function *via* VDR. Given this perspective, the comparison of the brain and serum 25(OH)D_3_ concentrations of the same individual might potentially provide an insight into understanding the relationships between neuropsychiatric diseases and the vitamin D status (sufficiency/deficiency).

**FIGURE 1:**
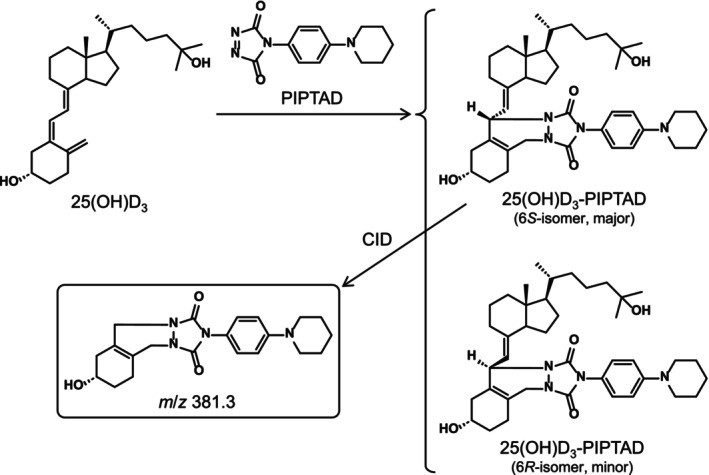
Derivatization reaction scheme of 25(OH)D_3_ with PIPTAD and structure of product ion from the derivative.

Some studies demonstrated that 25(OH)D_3_ was present in the rat (Xue et al. 2015) and mouse (Ahonen et al. 2014; Stephenson et al. 2023) brain tissues at quantifiable concentrations by liquid chromatography/tandem mass spectrometry (LC/MS/MS). 25(OH)D_3_ was also detected in a postmortem human brain sample (Fu et al. 2019). Xue et al. (2015) developed a method for quantifying 25(OH)D_3_ in the brain and serum of rats fed with different doses of vitamin D_3_ by LC/electrospray ionization (ESI)‐MS/MS combined with derivatization using 4‐phenyl‐1,2,4‐triazoline‐3,5‐dione (PTAD), a representative Cookson‐type reagent. With the aid of the derivatization, a clear peak derived from 25(OH)D_3_ was observed in the selected reaction monitoring (SRM) chromatogram of the brain sample; meanwhile, the nonderivatization method gave a peak with a low signal‐to‐noise ratio (S/N), which might be insufficient for identifying the peak as that derived from true 25(OH)D_3_ (Ahonen et al. 2014; Stephenson et al. 2023). Because the brain tissue is rich in oxysterols (Karu et al. 2011; Meljon et al. 2013) and some of which have the same molecular weights and similar physicochemical properties as 25(OH)D_3_, these oxysterols might interfere with the measurement of 25(OH)D_3_ in the brain tissue. We have demonstrated that derivatization is an effective approach to enhance the sensitivity and specificity for the LC‐ESI‐MS/MS quantification of lipophilic bioactive compounds in the brain (Higashi et al. 2024; Nishimoto‐Kusunose et al. 2023; Shibuya et al. 2024). Furthermore, we have developed several Cookson‐type reagents including 4‐(4‐dimethylaminophenyl)‐1,2,4‐triazoline‐3,5‐dione (DAPTAD) (Ogawa, Ooki, et al. 2013) and 4‐[4‐(1‐pipelidinyl)phenyl]‐1,2,4‐triazoline‐3,5‐dione (PIPTAD, Figure 1) (Takada et al. 2023). Among them, the derivatives with PIPTAD showed a stronger retention on the reversed‐phase LC than the derivative with other Cookson‐type reagents, which had a great advantage in separation from any interfering substances, as well as a high ESI‐MS/MS detectability (Takada et al. 2023). Due to these characteristics, PIPTAD was expected to work well for the analysis of 25(OH)D_3_ in the brain.

Xue et al. (2015) revealed that there was a strong positive correlation between the brain and serum 25(OH)D_3_ concentrations in rats fed with a greater‐than‐usual amount of vitamin D_3_, and the serum levels were always remarkably higher than the brain levels. This was a critical report demonstrating that the serum 25(OH)D_3_ concentration has a significant effect on its brain level. However, it is not yet known whether these results can be reproduced in the rats fed with a usual diet or in vitamin D deficiency‐model rats. As there has been an interest in the relationships between neuropsychiatric diseases and the vitamin D deficiency, a study using a vitamin D deficiency‐model rat becomes even more important. In the vitamin D deficiency‐model rat, the brain 25(OH)D_3_ is expected to be at less than ng/g tissue levels; therefore, an LC/ESI‐MS/MS method with an enhanced sensitivity and specificity is needed for reliably determining the brain 25(OH)D_3_ concentrations.

Based on this background information, the first objective of this study was to develop and validate a method for quantifying 25(OH)D_3_ in rat brain by the PIPTAD derivatization followed by LC/ESI‐MS/MS. The second one was the determination of the brain 25(OH)D_3_ concentrations in normal, fasted and bile duct‐ligated (BDL) rats to discuss the effect of the circulating (serum) 25(OH)D_3_ concentration on the brain 25(OH)D_3_ concentration.

## 2 Experimental

### 2.1 Chemicals, Reagents, and Materials

25(OH)D_3_ and [6,19,19‐^2^H_3_]‐25(OH)D_3_ [internal standard (IS)] were purchased from the FUJIFILM Wako Pure Chemical Corporation (Osaka, Japan) and IsoSciences (King of Prussia, PA, USA), respectively. The concentrations of the 25(OH)D_3_ working solutions (in ethanol) were 100, 200, 500, 1000, 2000, 5000 and 10,000 pg/mL. The IS working solution (in ethanol) with the concentration of 1000 pg/mL was also prepared. 3‐*Epi*‐25‐hydroxyvitamin D_3_ [3‐*epi*‐25(OH)D_3_] was purchased from the Cayman Chemical Company (Ann Arbor, MI, USA). 7α‐Hydroxycholestenone (Ogawa, Zhou, et al. 2013) and 4β‐hydroxy‐7‐dehydrocholesterol [4β(OH)‐7‐DHC] (Kawamoto et al. 2013) were gifts from Professor Takashi Iida (Nihon University, Tokyo). PIPTAD was synthesized in our laboratory by a known method (Takada et al. 2023). The LC/MS grade methanol and ammonium formate were purchased from the FUJIFILM Wako Pure Chemical Corporation and used for the mobile phase. A Puric‐α system (Organo, Tokyo) was used to prepare ultrapure water for the mobile phase. All other reagents and solvents were of analytical grade from the Tokyo Chemical Industry (Tokyo, Japan). A Strata‐X cartridge (60 mg adsorbent; Phenomenex, Torrance, CA, USA) was used immediately after successive flushing with ethyl acetate (1 mL), methanol (1 mL), and water (1 mL). An InertSep SI cartridge (500 mg adsorbent; GL Sciences, Tokyo) was used immediately after successive flushing with chloroform‐methanol (30:1, v/v, 4 mL) and chloroform (4 mL).

### 2.2 LC/ESI‐MS/MS

An LC/ESI‐MS/MS system comprised of a Shimadzu LC‐40D XS chromatograph and a Shimadzu LCMS‐8045 triple quadrupole mass spectrometer (Kyoto, Japan) was used in the positive‐ion mode. A Shimadzu SIL‐40C XS autosampler, DGU‐405 degasser, CTO‐40C column oven, and FCV‐20AH_2_ diversion valve were also parts of this system. Methanol‐10 mM of ammonium formate (4:1, v/v, isocratic mode) was used as the mobile phase and run through the Ascentis Express 90 Å C18 column (2.0 μm, 100 × 2.1 mm i.d.; Sigma‐Aldrich Japan, Tokyo) at the rate of 0.3 mL/min and the temperature of 40°C. The SRM was used for the quantification. The MS/MS conditions were as follows: interface voltage, 4.0 kV; Q1 prerod bias voltage, −20 V; Q3 prerod bias voltage, −26 V; collision energy, −31 eV; detector voltage, 1.76 kV; nebulizer gas (N_2_) flow rate, 3 L/min; drying gas (N_2_) flow rate, 5 L/min; desolvation line temperature, 250°C; heat block temperature, 400°C; and collision gas (Ar), 230 kPa. The SRM transitions were as follows: 25(OH)D_3_; *m*/*z* 659.6 → 381.3 and IS; *m*/*z* 662.6 → 384.3. LabSolutions software (version 5.118, Shimadzu) was used for the system control and data processing.

### 2.3 Collection of Brain and Serum Samples

All the rats (Wistar strain, male, 8 weeks old) were obtained from the Sankyo Labo Service Corporation (Tokyo). The normal and BDL rats were fed a conventional diet and filtered tap water ad libitum, whereas the fasted rats were given only water 24 h before the experiment. This diet contained vitamin D_3_ (2000 IU/kg) and no vitamin D_2_. The BDL rats were used 7 days after a common bile duct ligation operation (at 7 weeks old). BDL rat is a well‐known animal model of cholestasis (Johnstone and Lee 1976). It is also known that patients with cholestasis occur in conjunction with vitamin D deficiency due to impaired intestinal vitamin D absorption, which results from decreased secretion of bile acid into the duodenum (Chongthavornvasana et al. 2023). Based on these two observations, we thought that BDL rat could be a vitamin D deficiency‐model. Blood was taken from the postcava under isoflurane anesthesia. After standing at room temperature (*ca.* 20°C) for 30 min, the blood was centrifuged at 2000 × *g* (4°C, 30 min) to separate the serum. The brain (1.4–1.7 g) was eviscerated immediately after the blood collection and ultrasonically homogenized in saline (10 mL). The homogenate was stirred into ethanol (10 mL), then centrifuged at 1500 × *g* for 5 min. The supernatant was transferred to another test tube, then diluted with ethanol to adjust the concentration to 33.3 mg tissue/mL. This solution was described as the “brain extract” in this study. The serum sample and the brain extract were stored at −30°C before use. All animal care and use were approved by the Institutional Animal Care and Use Committee of Tokyo University of Science (approved no. Y22022).

### 2.4 Pretreatment Procedure of Brain Extract

The IS (10 pg) was added to the brain extract (1.5 mL, corresponding to 50 mg of brain tissue), and this mixture was diluted with water (3.5 mL), then passed through a Strata‐X cartridge. After washing the cartridge with water (2 mL) and 70% (v/v) methanol (1 mL), 25(OH)D_3_ and IS were eluted with ethyl acetate (1 mL). After evaporation of the solvent at 40°C under N_2_, the residue was immediately dissolved in chloroform (0.3 mL), then loaded on an InertSep SI cartridge. After washing the cartridge with chloroform (2 mL), 25(OH)D_3_ and IS were eluted with chloroform‐methanol (30:1, v/v, 2 mL). After evaporation of the solvent at 40°C under N_2_, PIPTAD (20 μg) in ethyl acetate (100 μL) was added to the residue and mixed. This mixture was left to stand at room temperature for 1 h. The reaction was terminated by adding ethanol (20 μL). After the solvent was evaporated, the residue was dissolved in the mobile phase (40 μL), 10 μL of which was subjected to LC/ESI‐MS/MS.

### 2.5 Calibration Curve

Endogenous 25(OH)D_3_ was removed from the brain extract (10 mL) by stirring overnight with activated charcoal (1.0 g). After centrifugation (2000 × *g*, 20°C, 10 min), the supernatant was used as the calibration curve matrix. The standard 25(OH)D_3_ (1.0, 2.0, 5.0, 10, 20 or 50 pg, corresponding to 20–1000 pg/g tissue) and IS (10 pg) were spiked in the calibration curve matrix (1.5 mL), then the resulting samples were pretreated and derivatized as described in the previous section. The peak area ratio [derivatized 25(OH)D_3_/IS, *y*] was plotted versus the 25(OH)D_3_ concentration (ng/g tissue, *x*) with a weighting of 1/*x* to construct the calibration curves.

### 2.6 Assay Precision and Accuracy

The intra‐assay and interassay precisions were assessed based on the relative standard deviations (RSDs, %) of five repeated measurements on 1 day and over 5 days, respectively. The intra‐assay and interassay accuracies were assessed based on the analytical recovery rates on 1 day and over 5 days, respectively. Two different brain samples were used for the precision and accuracy tests.

### 2.7 Recovery Rates During Solid‐Phase Extraction (SPE)

The recovery rates were calculated by dividing the peak area ratios [25(OH)D_3_/IS] of the prespiked samples by those of the postspiked samples (detailed in the Supporting Information).

### 2.8 Matrix Effect

The matrix effect was evaluated by the postextraction addition experiment (Thakare et al. 2016) (detailed in the Supporting Information).

### 2.9 Stability

The stability of 25(OH)D_3_ in the brain extract was assessed by comparing the measured values of five different rats before and after storage at room temperature (*ca.* 20°C) for 24 h (short‐term stability) and at −30°C for 5 months (long‐term stability). The stability of the derivatized 25(OH)D_3_ in the processed sample was assessed by comparing the measured values of five different rats before and after storage at 20°C in the autosampler for 24 h.

### 2.10 Quantification of 25(OH)D_3_ in Serum

The serum 25(OH)D_3_ was quantified by the LC/ESI‐MS/MS method developed and validated in our laboratories. Briefly, the serum (10 μL) was diluted, deproteinized, then derivatized with PIPTAD. [6,19,19‐^2^H_3_]‐25(OH)D_3_ was used as the IS. The validation test results were as follows: measurement range, 2.0–100 ng/mL; linearity, determination coefficient (*r* ^2^) ≥ 0.999; limit of detection (LOD, S/N = 3), 0.28 ng/mL; precision (intra‐assay and interassay RSDs), ≤ 5.0%; accuracy (analytical recovery rates), 95.3%–101.0%; and matrix effect, 98.7%. The details of the method will be reported elsewhere in the near future.

## 3 Results and Discussion

### 3.1 LC/ESI‐MS/MS Behavior of PIPTAD‐Derivatized 25(OH)D_3_

25(OH)D_3_ was derivatized with PIPTAD in the same way as reported in a previous study (Takada et al. 2023). A protonated molecule ([M + H]^+^, *m*/*z* 659.6) was produced from the derivatized 25(OH)D_3_ [25(OH)D_3_‐PIPTAD] as the base peak in the positive ESI‐MS. The collision‐induced dissociation (CID) of this protonated molecule gave a characteristic fragment ion at *m*/*z* 381.3 with a sufficient intensity as the result of cleavage of the C‐6–7 bond of the vitamin D skeleton (Figures 1 and S1a). Based on these results, the SRM mode with the transition of *m*/*z* 659.6 → 381.3 was used for quantifying 25(OH)D_3_. The derivatized IS showed similar ESI‐MS and ESI‐MS/MS behaviors.

**FIGURE 2:**
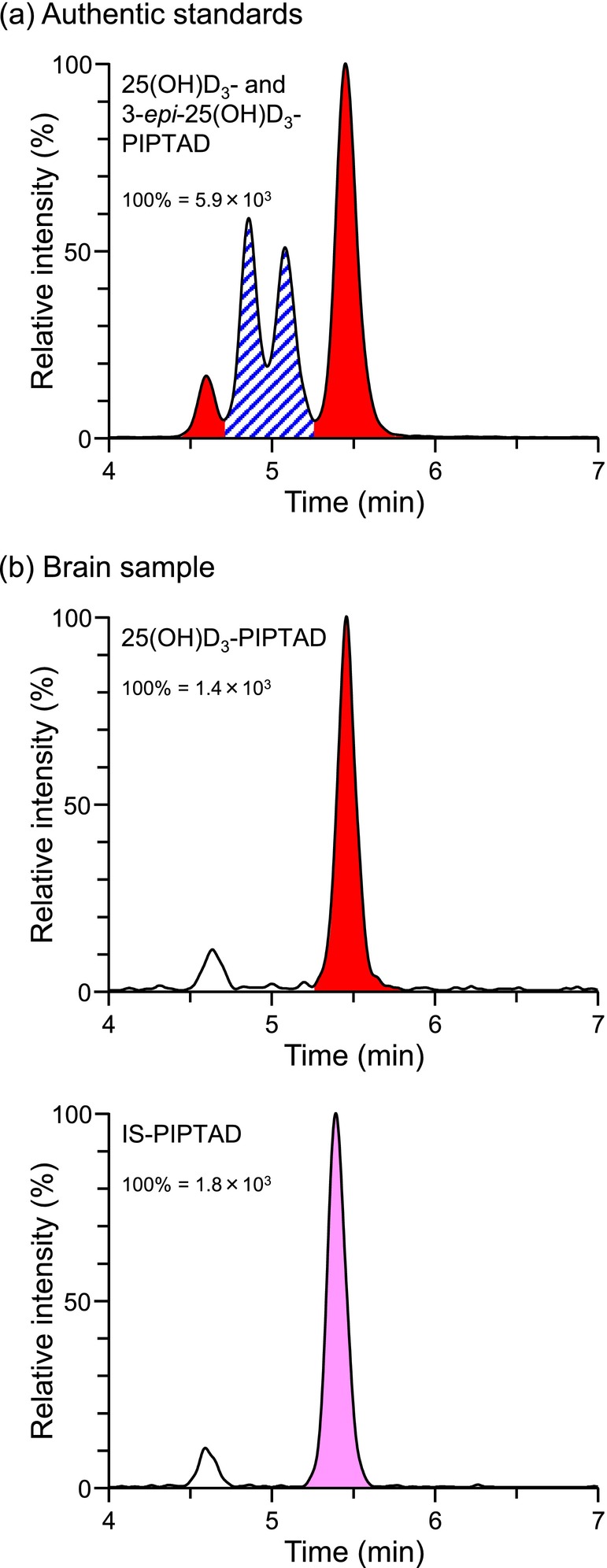
SRM chromatograms of 25(OH)D_3_, 3‐*epi*‐25(OH)D_3_ and IS as PIPTAD‐derivatives. (a) Authentic standards. A mixture of 25(OH)D_3_ and 3‐*epi*‐25(OH)D_3_ (100 pg each) was derivatized and dissolved in the mobile phase (100 μL), then 10 μL of which was injected into the LC/MS/MS. Red solid peaks: derivatized 25(OH)D_3_ and blue diagonal peaks: derivatized 3‐*epi*‐25(OH)D_3_. (b) Brain sample spiked with IS. The measured 25(OH)D_3_ concentration was 174 pg/g tissue.

25(OH)D_3_‐PIPTAD consisted of the 6*R*‐ and 6*S*‐isomers in the same way as the derivatives with other Cookson‐type reagents (Higashi and Shimada 2017). Therefore, 25(OH)D_3_‐PIPTAD gave two peaks at 4.6 and 5.5 min under the stated LC conditions (Figure 2). It has been reported that the 6*S*‐isomer is the major product in the reaction of 25(OH)D_3_ with various Cookson‐type reagents (Shimizu et al. 1995) and this isomer [retention time (*t*_R_), 5.5 min] was used for the quantification of 25(OH)D_3_ in this study.

For the quantification of 25(OH)D_3_ in biological samples, one of the complicated problems is the potential interference from 3‐*epi*‐25(OH)D_3_, leading to overestimation of the true 25(OH)D_3_ concentrations (Bailey et al. 2013; Higashi and Shimada 2017). As shown in Figure 2a, the PIPTAD‐derivatized 3‐*epi*‐25(OH)D_3_ was eluted at 4.9 and 5.1 min as the twin peaks (we could not determine which is the 6*R*‐ or 6*S*‐isomer) and satisfactorily separated from 25(OH)D_3_‐PIPTAD; the resolution value was calculated to be 1.51 based on the widths at half height of the neighboring peaks (*t*_R_ 5.1 and 5.5 min). This good separation became possible due to the combination of the PIPTAD derivatization and use of the Ascentis Express 90 Å C18 column packed with superficially porous particles. Thus, the developed method had a tolerance for interference from 3‐*epi*‐25(OH)D_3_.

The effect of the PIPTAD derivatization on the ESI‐MS/MS detectability of 25(OH)D_3_ was evaluated by comparing the S/Ns before and after the derivatization. In this experiment, 2.5 fmol each of the intact (underivatized) and derivatized 25(OH)D_3_ were injected into the LC/ESI‐MS/MS. The S/N value of 25(OH)D_3_‐PIPTAD was 203 ± 12 [mean ± standard deviation (SD), *n* = 3], which was 50 times greater than that of the intact 25(OH)D_3_ (S/N, 4.1 ± 0.3). As will be shown later, the trace 25(OH)D_3_ in the brain could be quantified due to this detectability enhancing effect.

### 3.2 Discrimination Between 25(OH)D_3_ and Oxysterols by PIPTAD Derivatization

**FIGURE 3:**
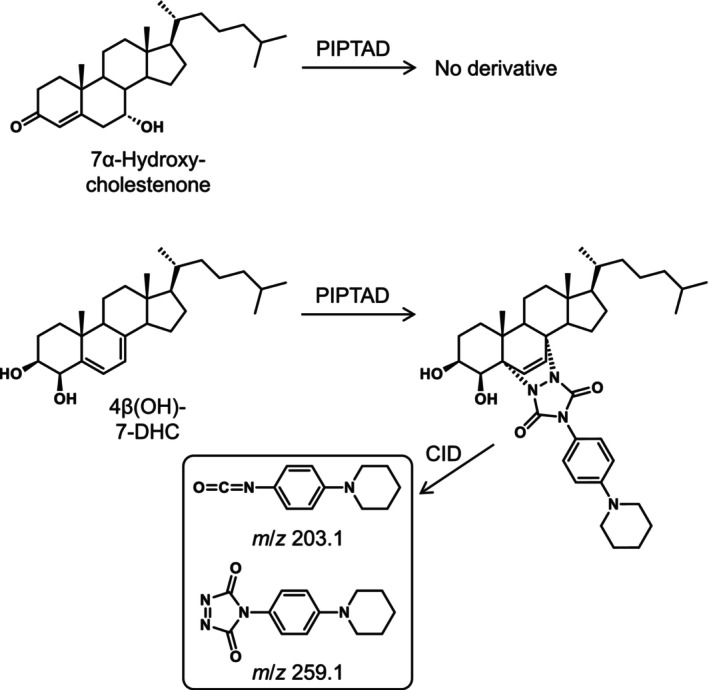
Reaction of 7α‐hydroxycholestenone and 4β(OH)‐7‐DHC with PIPTAD. Structures of product ions from the derivatized 4β(OH)‐7‐DHC are also shown.

As mentioned in Section 1, oxysterols are abundant in the brain tissue (Karu et al. 2011; Meljon et al. 2013). Monohydroxycholest‐4‐ene‐3‐ones, such as 7α‐hydroxycholestenone (Mutemberezi et al. 2016), and monohydroxy‐7‐dehydrocholesterols, such as 4β(OH)‐7‐DHC (Meljon et al. 2013), are potential endogenous oxysterols and have the same molecular weights as 25(OH)D_3_; therefore, the interference from these oxysterols was of concern for the quantification of 25(OH)D_3_ in the brain. However, the monohydroxycholest‐4‐ene‐3‐ones did not undergo a reaction with PIPTAD due to a lack of the *s*‐*cis*‐diene (Figure 3). Although the monohydroxy‐7‐dehydrocholesterols produce derivatives after the reaction with PIPTAD, the derivatives showed a different fragmentation pattern from 25(OH)D_3_‐PIPTAD during the CID (Figures 3 and S1b). As demonstrated in the prior section, 25(OH)D_3_‐PIPTAD gave the product ion at *m*/*z* 381.3 resulting from cleavage of the C–6–7 bond of the vitamin D skeleton (Figure 1), whereas 4β(OH)‐7‐DHC‐PIPTAD did not give this product ion but gave the product ions at *m*/*z* 203.1 and 259.1, which were derived from the PIPTAD moiety. Accordingly, when the SRM of *m*/*z* 659.6 → 381.3 was used, 25(OH)D_3_‐PIPTAD could be specifically detected even in the presence of a large amount of isomeric oxysterols. Thus, the PIPTAD derivatization significantly enhanced the specificity for the brain assay.

### 3.3 Pretreatment of Brain Extract

The brain extract corresponding to 50 mg of brain tissue was sequentially purified using reversed phase and normal phase SPE cartridges. The recovery rate of 25(OH)D_3_ during the sequential SPE process was 63.2 ± 1.4% (mean ± SD from five different brain samples). Thus, 25(OH)D_3_ was acceptably and reproducibly recovered from the brain extract sample. The elution fraction was derivatized with PIPTAD after evaporation to dryness.

The typical chromatograms of the pretreated brain sample are shown in Figure 2b. The peak of the major isomer of 25(OH)D_3_‐PIPTAD was clearly observed (*t*_R_ 5.5 min). No significant matrix effect was observed for 25(OH)D_3_ (100.3 ± 1.5%, mean ± SD, *n* = 5) and IS (95.2 ± 2.8%) despite the complexity of the brain matrix. This result demonstrated that the two‐step SPE‐based pretreatment procedure worked well to remove the brain‐derived substances that cause ion suppression/enhancement, although the procedure was time‐consuming. The LOD (S/N = 3) was calculated to be 5 pg/g tissue based on the measured concentrations and S/N values of the brain samples of the BDL rats.

### 3.4 Calibration Curve

A good linearity (*r* ^2^ > 0.999) and reproducibility (RSD of slope, 1.7%) were obtained within the range of 20–1000 pg/g tissue; the regression formula was *y* = (4.5657 ± 0.0794) × 10^−3^ *x* + (0.0098 ± 0.0190) × 10^−3^ (the slope and *y*‐intercept were given as the mean ± SD, *n* = 5).

### 3.5 Assay Precision and Accuracy

As shown in Table 1, the maximum RSD value in the intra‐assay and interassay precision tests was 4.0%, which was much lower than the generally accepted criterion (≤ 15%). The analytical recovery rates ranged from 97.7% to 101.2%, which fell within the guidelines (100 ± 15%). Thus, a highly precise and accurate method was established.

**TABLE 1:** Assay precision and accuracy.

	Brain A	Brain B
Intra‐assay		
Precision		
Measured (pg/g tissue)^a^	66 ± 1	175 ± 7
RSD (%)	1.5	4.0
Accuracy (%)		
+ 50 pg/g tissue spiked^a^	97.8 ± 4.4	Not examined
+100 pg/g tissue spiked^a^	99.3 ± 3.9	98.4 ± 3.5
+ 200 pg/g tissue spiked^a^	Not examined	101.2 ± 4.2
Interassay		
Precision		
Measured (pg/g tissue)^a^	67 ± 1	180 ± 7
RSD (%)	1.5	3.9
Accuracy (%)		
+ 50 pg/g tissue spiked^a^	98.6 ± 2.3	Not examined
+ 100 pg/g tissue spiked^a^	101.0 ± 1.4	99.4 ± 3.4
+ 200 pg/g tissue spiked^a^	Not examined	97.7 ± 2.5

^a^ Mean ± SD (*n* = 5).

### 3.6 Stability

There were no significant changes in the 25(OH)D_3_ concentration in the brain extract during the short‐term storage (at 20°C for 24 h; 98.7 ± 2.6%, mean ± SD, *n* = 5) and long‐term storage (at −30°C for 5 months; 98.3 ± 5.1%). The processed samples were also stable at 20°C for at least 24 h (100.6 ± 5.3%).

### 3.7 Determination of 25(OH)D_3_ Concentrations in the Rat Brain and Effect of the Serum Concentrations on the Brain Concentrations

**FIGURE 4:**
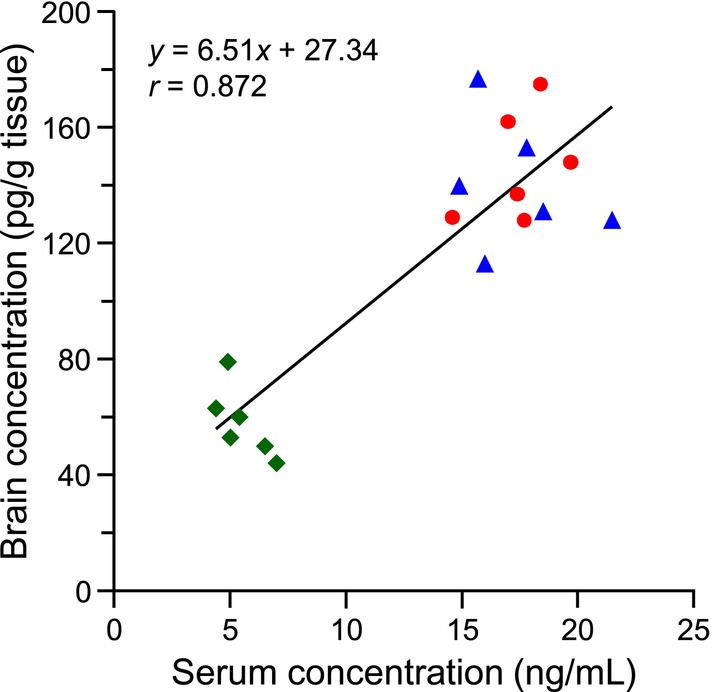
Scatter diagrams to show the distribution of the brain and serum 25(OH)D_3_ concentrations and the correlation between the two. Red circle; normal rat, blue triangle; fasted rat and green diamond; BDL rat (*n* = 6 each).

Figure 4 shows the distribution of the brain and serum 25(OH)D_3_ concentrations of the normal, fasted, and BDL rats (*n* = 6 each) and the correlation between the brain and serum levels. The mean concentrations with the SD values were 147 ± 19, 140 ± 22, and 58 ± 12 pg/g tissue for the normal, fasted, and BDL rats, respectively. The serum 25(OH)D_3_ concentrations were extremely higher than the brain concentrations: 17.5 ± 1.7, 17.4 ± 2.4, and 5.5 ± 1.0 ng/mL (mean ± SD) for normal, fasted, and BDL rats, respectively. The brain 25(OH)D_3_ concentration‐to‐serum 25(OH)D_3_ concentration ratios (B/S ratios) ranged from 1/62 to 1/167. The circulating 25(OH)D_3_ exists in three forms with the approximate ratio of 85%–90% as the vitamin D binding protein (DBP)‐bound form, 10%–15% as the albumin‐bound form, and 0.03% or less in the protein‐unbound (free) form (Ishimine et al. 2022). DBP‐ and albumin‐bound 25(OH)D_3_ cannot enter the brain because proteins hardly cross the blood–brain barrier, whereas free fraction of the circulating 25(OH)D_3_ can cross the blood–brain barrier by passive diffusion due to its high hydrophobicity and low molecular weight. Given this extremely low rate (0.03%) of the free form, the above‐mentioned low B/S ratio (1/62–1/167) was a logical outcome.

For quantifying the brain 25(OH)D_3_, the small residual blood in the brain tissue was of concern because it was impossible to completely remove the blood from the brain sample. This was one of the limitations of this study. If the DBP concentration is significantly lower than the 25(OH)D_3_ concentration in the brain sample, the possibility that blood contamination is a major cause of the detection of 25(OH)D_3_ in the brain sample can be excluded. The comparison of the DBP and 25(OH)D_3_ concentrations in the brain sample merits further research.

In our previous study (Shibuya et al. 2024), the concentrations of bile acids were determined in the brain samples collected in the similar way as this study and compared to their serum concentrations. The B/S ratios of cholic acid and lithocholic acid in normal rats (*n* = 12) were 1/39–1/96 and 1/17–1/56, respectively. If 25(OH)D_3_ in the residual blood was the main source of 25(OH)D_3_ detected in the brain sample, its B/S ratios would be in the same range as those of the bile acids. However, the B/S ratios of 25(OH)D_3_ (1/62–1/167) significantly differed from those of the bile acids. Based on these observations, we inferred that the blood contamination was not the major cause of the detection of 25(OH)D_3_ in the brain sample.

A 24‐h fasting had virtually no influence on the brain and serum 25(OH)D_3_ concentrations; this could be due to the relatively long half‐life (2–3 weeks) of the circulating 25(OH)D_3_ (Holick 2009). On the other hand, the bile duct ligation treatment significantly decreased the 25(OH)D_3_ concentrations in both the brain and serum. In the BDL rats, bile acids, which are essential for absorption of the lipid‐soluble nutrients including vitamin D, are not secreted into the duodenum; this deficient secretion of bile acids causes the decreased absorption of vitamin D_3_ from their diet. Even the fur‐bearing animals can synthesize vitamin D_3_ on their skin when they are exposed to UV‐B (Carpenter and Zhao 1999; Hymøller and Jensen 2010). However, the rats used in this study were housed under a conventional LED lamp, which does not emit UV‐B, during the light period (08:00–20:00 h). Therefore, it is presumed that the photochemically synthesized vitamin D_3_ on their skin is very limited, and the main source of vitamin D_3_ is their diet for the rats used in this study. Therefore, the BDL rat can be the vitamin D deficiency‐model. These rats were kept in a state of bile duct ligation for 7 days, and in the meantime, the serum 25(OH)D_3_ levels decreased, which would cause the subsequent decreased brain 25(OH)D_3_ levels. Thus, our study ensured that the serum 25(OH)D_3_ concentrations have a significant effect on the brain 25(OH)D_3_ levels. When all the rats were lumped together (*n* = 18), there was a positive correlation between the brain and serum 25(OH)D_3_ concentrations with a correlation coefficient of 0.872 (Figure 4); this result agreed well with that of the previous study (Xue et al. 2015).

## 4 Conclusion

The LC/ESI‐MS/MS method was developed and validated for quantifying 25(OH)D_3_ in the rat brain tissue. The PIPTAD derivatization enhanced the assay sensitivity and specificity. Based on the developed method, the brain 25(OH)D_3_ was quantified in the normal, fasted, and BDL rats. The brain 25(OH)D_3_ concentrations were slightly over 100 pg/g tissue under the normal conditions and were extremely lower than the serum concentrations. The bile duct ligation caused the decreased serum 25(OH)D_3_ level, which produced the subsequent decreased brain 25(OH)D_3_ level. These results may provide evidence that the serum 25(OH)D_3_ concentration has a significant effect on its brain level.

## Conflicts of Interest

The authors declare no conflicts of interest.

## Supporting information


**Figure S1.** Product ion spectrum of (a) PIPTAD‐derivatized 25(OH)D_3_ and (b) 4β(OH)‐7‐DHC.

## Data Availability

Data will be made available upon request.
