# Supplementary material for: Determination of 25‐Hydroxyvitamin D3 in Rat Brain by Derivatization‐Assisted LC/ESI‐MS/MS
Source: Biomed Chromatogr. 2025 May 22;39(7):e70119. doi: 10.1002/bmc.70119 (PMC12097965; doi:10.1002/bmc.70119)
Supplement: Supplementary file 1 — Figure S1. Product ion spectrum of (a) PIPTAD‐derivatized 25(OH)D3 and (b) 4β(OH)‐7‐DHC. [file BMC-39-e70119-s001.docx]

*Biomedical Chromatography*

RESEARCH ARTICLE

**Determination of 25-Hydroxyvitamin D_3_ in Rat Brain by Derivatization-Assisted LC/ESI-MS/MS**

Toma Shibuya^1^ | Fuwari Shishikura^1^ | Natsuki Yoshida^1^ | Shoujiro Ogawa^2^ | Tatsuya Higashi^1^

^1^ Faculty of Pharmaceutical Sciences, Tokyo University of Science, 6-3-1 Niijuku, Katsushika, Tokyo 125-8585, Japan | ^2^ Faculty of Pharmacy and Pharmaceutical Sciences, Fukuyama University, 1 Sanzo, Gakuen‑cho, Fukuyama 729‑0292, Japan

Correspondence: Tatsuya Higashi (higashi@rs.tus.ac.jp)

**Samples for Evaluating Recovery Rates during SPE**

Pre-spiked sample: The activated charcoal-treated brain extract (1.5 mL, *n* = 5) was spiked with 25(OH)D_3_ (10 pg), then pretreated as already described. After the addition of the IS (10 pg), the sample was derivatized and subjected to LC/ESI-MS/MS.

Post-spiked sample: The activated charcoal-treated brain extract (1.5 mL, *n* = 5) was pretreated as already described. After the addition of 25(OH)D_3_ and IS (10 pg each), the sample was derivatized and subjected to LC/ESI-MS/MS.

**Samples for Evaluating Matrix Effect**

Standard sample; 25(OH)D_3_ (100 pg) was derivatized and dissolved in ethanol (100 μL). An aliquot (10 μL) of this ethanolic solution was evaporated, then the residue was dissolved in the mobile phase and injected into the LC/ESI-MS/MS (*n* = 5).

Matrix sample; the brain extract was pretreated as previously described. To the obtained residue containing the brain matrix, 10 μL of the above standard sample solution was added. After evaporation of the solvent, the residue was dissolved in the mobile phase and injected into the LC/ESI-MS/MS (*n* = 5).

**FIGURE S1** | Product Ion Spectrum of (a) PIPTAD-Derivatized 25(OH)D_3_ and (b) 4β(OH)-7-DHC.
